# Numerical simulation and experimental verification of the velocity field in asymmetric circular bends

**DOI:** 10.1038/s41598-024-64978-6

**Published:** 2024-06-17

**Authors:** Lu Jia, Yongzhong Zeng, Xiaobing Liu, Chao Peng, Dali Li, Fei Liu, Lindong He

**Affiliations:** 1 Key Laboratory of Fluid and Power Machinery, Ministry of Education, Xihua University, Chengdu, 610097 China; 2 Sichuan Southwest Vocational College of Civil Aviation, Chengdu, 610039 China; 3 Tianfu College of Southwestern University of Finance and Economics, Chengdu, 621000 China

**Keywords:** Metrology, Flow measurement, Coefficient of variation, CFD numerical simulation, Velocity distribution, Asymmetric circular pipe, Environmental sciences, Space physics, Energy science and technology, Engineering, Physics

## Abstract

To address the measurement accuracy challenges posed by the internal flow complexity in atypical circular bend pipes with short turning sections and without extended straight pipe segments, this study designed an experimental circular “S”-shaped bent pipe with a diameter of 0.4 m and a bending angle of 135°. Numerical analysis was used to determine the stable region for velocity distribution within the experimental segment. Furthermore, a novel evaluation method based on the coefficient of variation was proposed to accurately locate the optimal position for installing thermal mass flow meters on the test cross section. Additionally, a formula for calculating the pipeline flow rate based on velocity differences was derived. This formula considers pipeline flow as the dependent variable and uses the velocity at two points in the test cross section as the independent variable. Experimental validation on a primary standard test bench demonstrated that the flow rate calculated by this method had an error controlled within 0.625% compared to the standard flow rate, thus effectively verifying the method’s high accuracy and engineering applicability. This research provides a new testing methodology and practical basis for flow measurement in complex pipeline systems, offering significant guidance for research and applications in related fields.

## Introduction

Flow measurement technology is widely used in industrial fields, such as petroleum, chemical, and energy fields. Most flow meters currently in widespread use measure volumetric flow rates. However, when external environmental conditions, such as temperature and pressure, change, the volumetric flow rate needs to be converted into a value under standard or specified conditions. This conversion can be challenging to achieve in real time; therefore, directly obtaining the mass flow rate of the fluid is more desirable^1–4^. To address this issue, researchers have designed various types of flow sensors^5,6^. Thermal mass flow meters consist of a heater and one or more temperature sensors, which measure flow by analysing the heat exchange between the fluid and the heat source. These devices are favoured for their simple operating principles, streamlined structural design, minimized pressure drops, and compact components^7^.

When measuring flow in large-diameter pipelines, traditional pipeline flow meters are not ideal due to their substantial size, significant pressure drop, and high energy consumption^8^. Instead, devices such as averaging tubes, thermal mass flow meters, and ultrasonic flow meters for gases overcome these drawbacks. They are favoured for their compact structure, ease of installation, and maintenance simplicity^9,10^. In the design of ventilation duct systems, the spatial constraints of plant sites and the varied configurations of duct accessories prevent airflow within ducts from achieving a fully developed turbulent state^11,12^.

In large pipe flow measurements, different methods are needed to enhance the measurement accuracy. Liu^13^, in a large-scale air duct flow measurement system, used a rectifier grate to straighten the flow field, reducing airflow blockage and pressure loss, and adopted a matrix layout of multipoint pitot tube sensors to scientifically arrange flow velocity measurement points within large pipes. Zhang^14^ used the velocity-area method for flow measurement in rectangular enclosed ducts by measuring the velocity first and then multiplying it by the cross-sectional area to obtain the flow rate. Mao^15^researched large pipe gas flow measurements by employing insertion flow instruments, which can be classified into measuring point speed, measuring line speed, and measuring multipoint speeds on the cross section. Under laboratory conditions, they proposed a correction method for uniform flow fields, establishing a uniform field where the flow rate can be determined by measuring the velocity at a single point, thereby simplifying the testing procedure^16^. Sai^17^and others studied the application of combined total pressure tubes in the flow measurement of large pipes with diameters greater than 1 m. Through numerical simulations of different sampling methods and quantities of total pressure holes, they found that using Chebyshev’s method with four pairs of holes in a single arrangement provided the best results, with relatively small measurement errors. Research on Coriolis mass flowmeters has focused mainly on the structure of the measuring tube to improve instrument accuracy, stability, and sensitivity; increase tube deflection; improve stress distribution; reduce fatigue failure; and enhance vibration resistance^18^. However, due to challenging on-site measurement conditions, the flow measurements of gases with large cross-sections and low velocities often fail to meet the minimum requirements for accurate measurements in straight duct sections. Consequently, using these measurement devices can lead to uncertainties in data accuracy, making these flow rates difficult to assess. To achieve a more stable distribution of flow velocities, the fluid must pass through a certain length of straight duct after entering the pipeline, typically at least 15 times the diameter of the pipe^19^.

In summary, based on different application requirements and environmental conditions, an appropriate type of flow sensor and measurement method can be selected to achieve accurate flow measurements. Therefore, this paper presents a method to determine the placement of measurement points and a formula to calculate the average flow rate at the cross-section in the flow measurement of a 0.4 m diameter, 135° circular “S” shaped bent pipe, derived from numerical simulation analysis. This method offers more precise measurements and can effectively meet the flow measurement needs in practical engineering projects. This study also provides methodological guidance for future flow measurements in atypical circular bend pipes using thermal mass flow meters.

## Numerical analysis of the flow field at the test cross-section of atypical circular bent pipes

### 3D Geometric modelling

**Figure 1:**
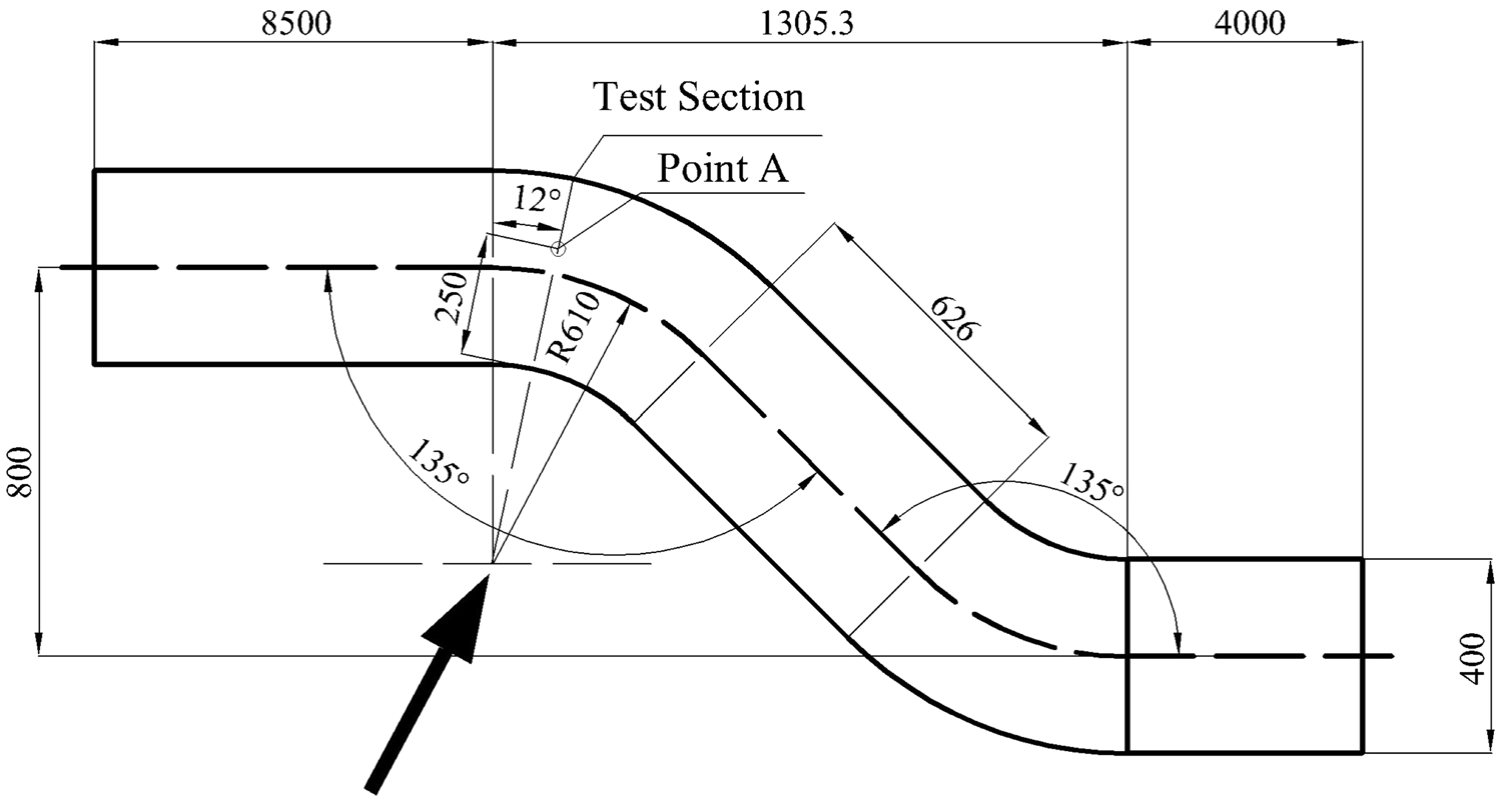
Fabrication diagram of the asymmetric circular bend test section.

The subject of this study is an atypical circular bent pipe with a diameter of 0.4 m, as shown in Fig. 1. The test cross section, indicated by the arrow in the figure, is the specific location of the study. This research focuses on analysing the velocity distribution at the test cross section. CFD technology facilitates a detailed numerical analysis of this atypical circular bent pipe, and the flow field can be visually displayed.

**Figure 2:**
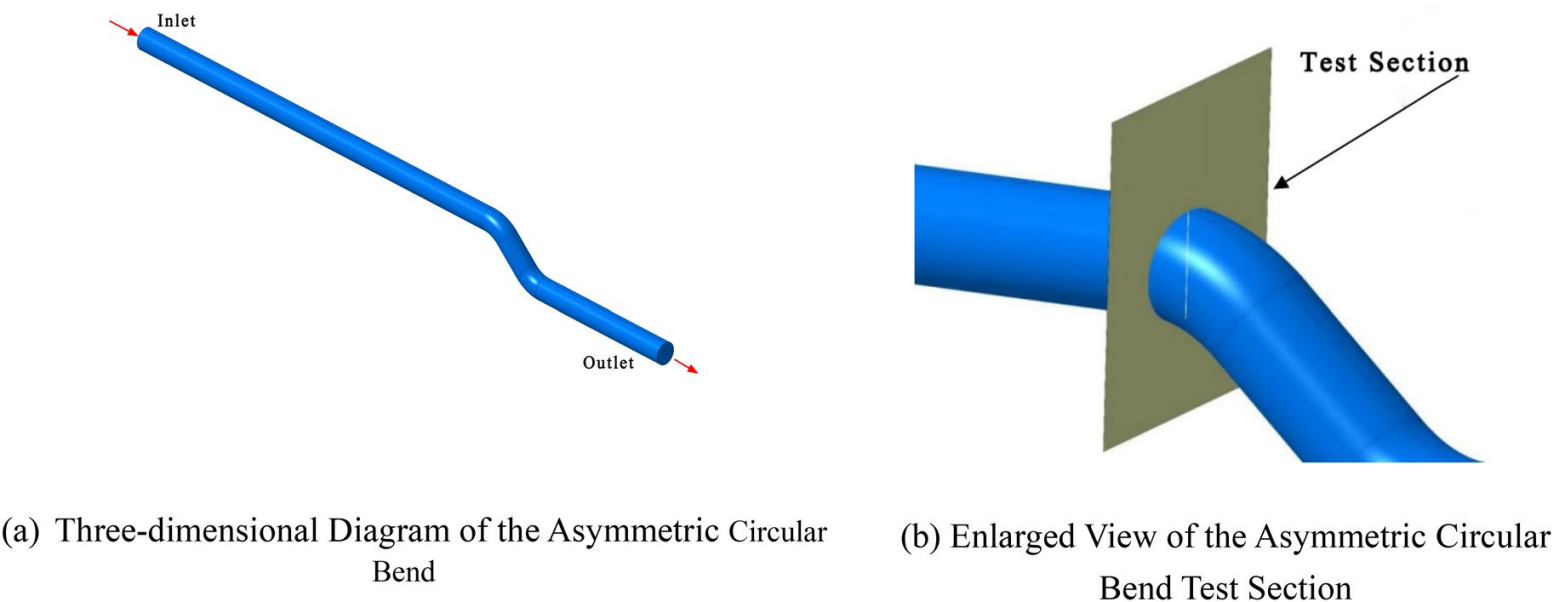
Geometric model of the asymmetric circular bend.

An atypical circular bend pipe with a diameter of 0.4 m was geometrically modelled. To achieve a more uniform and stable flow field distribution in the inlet and outlet sections, these sections were appropriately extended. The length from the inlet section to the test cross-section was set at 20D, which is 8 m, as shown in Fig. 2. On the test cross-section, a line segment passing through the diameter was drawn, and two test points were selected on this line (based on the coefficient of variation) for data measurement.

### Fluid mechanics theoretical analysis

Fluid flow is subject to the fundamental laws of physics, including crucial conservation principles: conservation of mass, momentum, and energy. In cases involving the mixing or interaction of distinct components, the system also adheres to the conservation of compositions. Additionally, turbulent flow scenarios require adherence to supplementary turbulent transport equations.

The mass conservation equation is defined as follows:

1 $$\frac{{\partial \left( {\rho u_{i} } \right)}}{{\partial x_{i} }} = 0$$

where $\rho$ is the density of fluid; $u_{i}$ is the flow velocity in direction *i*; and *i*-1, 2, 3, and $x_{i}$ represent three coordinate directions.

The momentum conservation equation is defined as follows:

2 $$\rho u_{i} + \frac{{\partial \left( {\rho u_{i} u_{j} } \right)}}{{\partial x_{j} }} = - \frac{\partial p}{{\partial x_{j} }} + \frac{\partial }{{\partial x_{j} }}\left( {\mu \left( {\frac{{\partial u_{i} }}{{\partial x_{j} }} + \frac{{\partial u_{j} }}{{\partial x_{i} }}} \right)} \right) + g_{i}$$

where $p$ is the static pressure; $\mu$ is the molecular viscosity; $g_{i}$ is the mass force in the *i* direction; and* j* − 1, 2, 3.

The subject of this study is air, which has a high Reynolds number in its flow, making turbulence effects nonnegligible. Therefore, the most commonly used Reynolds-averaged Navier‒Stokes (RANS) equations combined with the shear stress transport (SST)$k - \omega$ turbulence model are chosen to simulate the flow phenomenon in this engineering context^20^. Its governing equations are defined as follows:

3 $$\rho k + \frac{\partial }{{\partial x_{i} }}\left( {\rho ku_{i} } \right) = \frac{\partial }{{\partial x_{i} }}\left( {\Gamma_{k} \frac{\partial k}{{\partial x_{i} }}} \right) + G_{k} - Y_{k}$$

4 $$\rho \omega + \frac{\partial }{{\partial x_{i} }}\left( {\rho \omega u_{i} } \right) = \frac{\partial }{{\partial x_{i} }}\left( {\Gamma_{\omega } \frac{\partial k}{{\partial x_{i} }}} \right) + G_{\omega } - Y_{\omega }$$

In these equations, *k* is the turbulent kinetic energy, $\omega$ is the specific dissipation rate, $G_{K}$ represents the generation of turbulence kinetic energy due to mean velocity gradients, $G_{\omega }$ represents the generation of $\omega$, $\Gamma_{K}$ and $\Gamma_{\omega }$ represent the effective diffusivity of *k* and $\omega$, and $Y_{K}$ and $Y_{w}$ represent the dissipation *k* of $\omega$ and due to turbulence.

### Computational boundary conditions

When selecting the positions of the inlet and outlet boundaries, the flow might not have reached a fully developed state if the flow inlet and outlet boundaries are too close to solid obstacles or too close to areas of unstable flow, leading to significant errors. To obtain accurate results, the outlet boundary should generally be located at least 10 times the height of the last obstacle. For higher precision requirements, the sensitivity of the simulation results to the outlet position at different distances also needs to be studied to ensure that the internal simulation is not affected by the choice of outlet location.

Therefore, for the calculations, 14.904, 22.017, 26.224, and 29.076 m·s^−1^ were chosen for the four different operating conditions. For each condition, the flow rate was recorded three times, and the average velocity was then calculated, as shown in Table 1.

**Table 1:** Computational boundary conditions.

Operating conditions	Flow points (%)	Standard flow rate (m^3^ h^−1^)	Velocity (m s^−1^)
1	25%Q_max_	3365.3921	14.904
		3365.4858	
		3365.4405	
2	50%Q_max_	6714.3185	22.017
		6713.9599	
		6714.3939	
3	75%Q_max_	9669.8964	26.224
		9669.0948	
		9669.5682	
4	100%Q_max_	13,366.2855	29.076
		13,364.6853	
		13,366.1036	

### Mesh division

**Figure 3:**
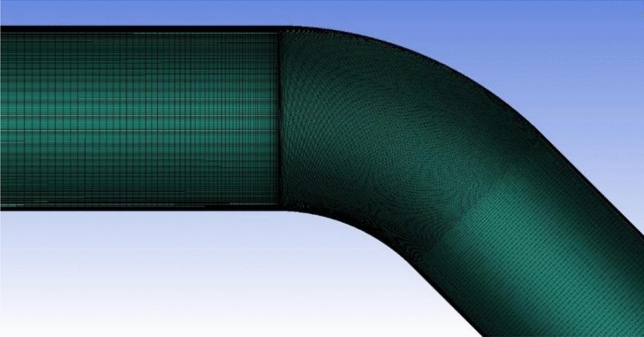
Mesh division of the asymmetric circular pipe.

A fully structured mesh is used to grid the geometry, as shown in Fig. 3, with grid refinement near the test section to ensure that the flow in this region is captured in detail. In numerical calculations, the number of grids significantly impacts the convergence and accuracy of the computation. However, a higher number of grids does not necessarily equate to higher computational accuracy. Excessive grids can lead to an overwhelming workload for the computer, increasing computational time and cost without a corresponding improvement in accuracy. Therefore, conducting a grid independence analysis is essential before performing numerical calculations to determine the appropriate number of grids for the computation.

**Figure 4:**
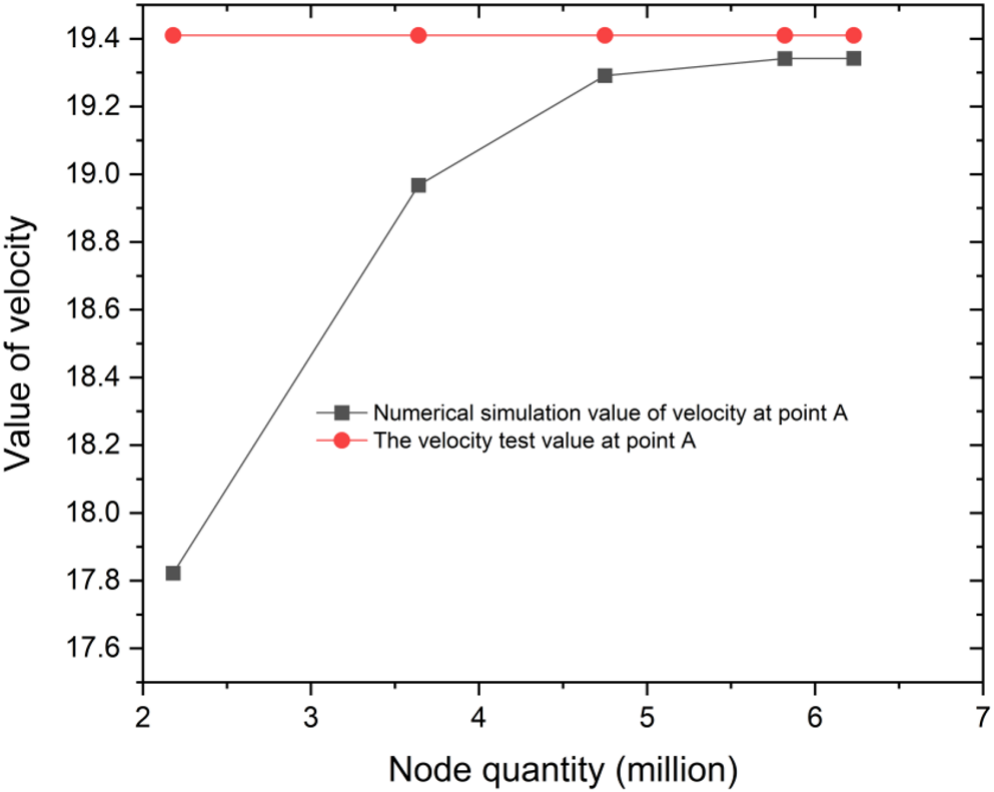
A grid independence analysis.

For numerical validation, the average velocity at the test section point A was used as the benchmark, as shown in Fig. 1. Five different sets of computational grids were used, totalling 2.18 million, 3.64 million, 4.75 million, 5.82 million, and 6.23 million grids, as shown in Fig. 4. The average velocity gradually increased with increasing grid count from 2 to 6 million, indicating a significant impact of the grid number on the calculations. However, once the grid count reached 6 million, the average velocity no longer increased with additional grids, and the curve stabilized. This finding suggests that further increases in grid numbers would no longer affect the results. Therefore, a grid count of 5.82 million was chosen for the final numerical computations.

### Numerical calculation parameters

The computational boundary conditions and computational models are shown in Table 2.

**Table 2:** Fluent Computational Parameters.

Solver	Turbulence model	Fluid properties	Boundary conditions	First layer wall mesh size (mm)
Couple	SST *k-ε*	Temperature 20 °C Pressure1atm Density 1.205 kg·m^−3^	Velocity Inlet Atmospheric pressure outlet No-Slip Wall	0.01

### Analysis of the calculation results for the test cross-section of atypical circular bent pipes

**Figure 5:**
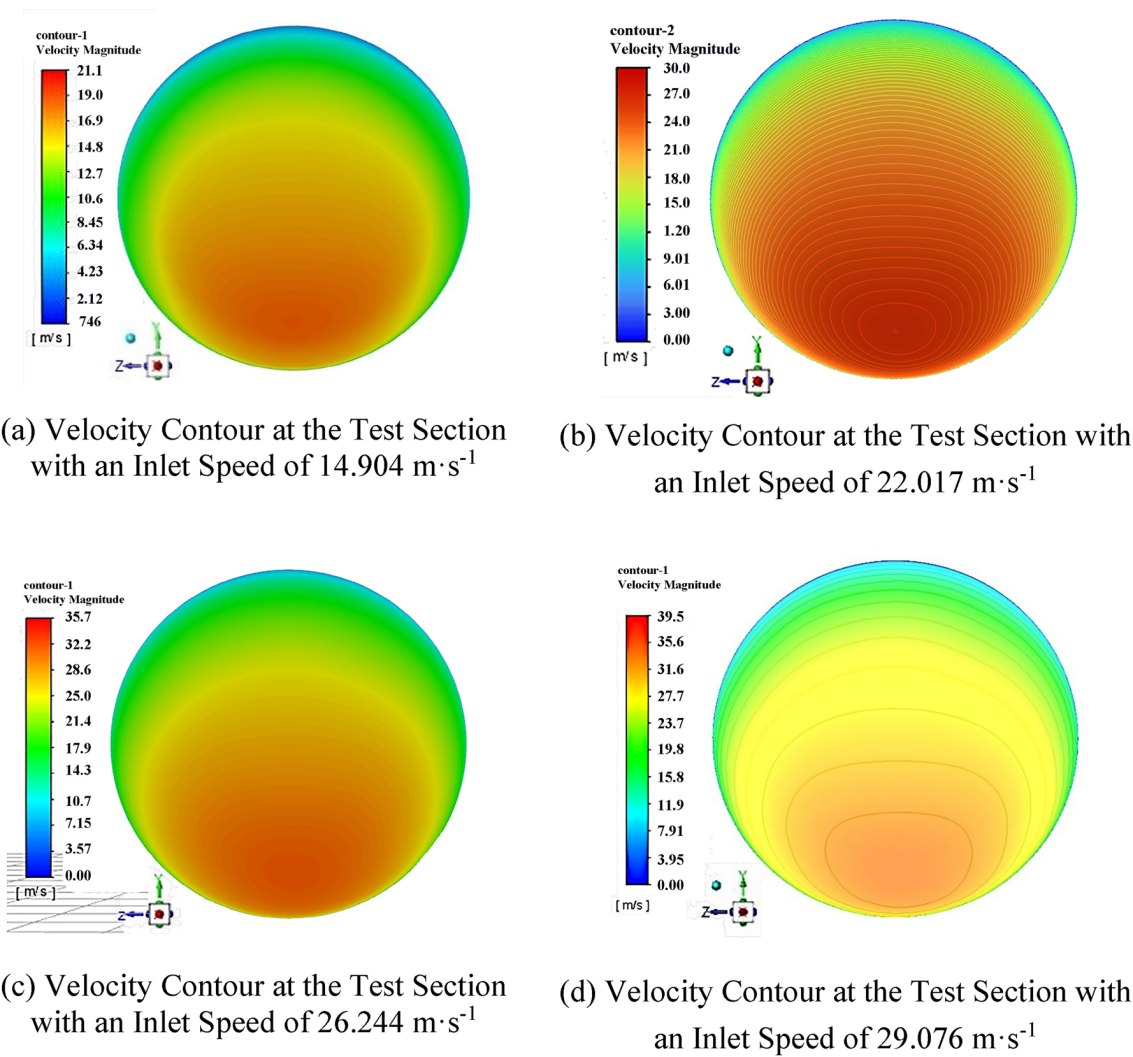
Velocity coefficient distribution contours at the test cross-section.
  Overall, the structure of the flow field is similar under different flow rate conditions, with high-speed and low-speed areas existing on both sides of the flow field. However, a stable region exists between these areas. This stable region can be determined by calculating the coefficient of variation of the relative velocity distribution under different conditions and identifying the position where this coefficient is minimal.
(2) Determination of velocity meter measurement point locations in a 0.4 m diameter circular bent pipe
  To quantitatively compare the distributions of these discrete velocity values, the coefficient of variation, *C*_v,_ is used for calculation and comparison. *C*_v_ is the percentage of the standard deviation of a set of data relative to its mean value and is a relative indicator for measuring the dispersion of data, representing a relative measure of variation. The coefficient of variation is typically calculated using the standard deviation as follows:
  5 $$\begin{gathered} \hfill \\ C_{V} = \sqrt {\frac{{\sum\limits_{i = 1}^{i = 4} {\left( {\frac{{v_{i} }}{{v_{m} }} - \overline{{\left( {\frac{v}{{v_{m} }}} \right)}} } \right)^{2} } }}{{4\overline{{\left( {\frac{v}{{v_{m} }}} \right)}} }}} \hfill \\ \end{gathered}$$
  In the formula, *v*_*i*_*/v*_*m*_ represents the relative flow velocity at each measurement point under different operating conditions; i = 1…4 corresponds to the four operating conditions of 14.904, 22.017, 26.224, and 29.076 m·s^−1^, respectively;* v*_*i*_ is the flow velocity at each measurement point on the test cross-section under different conditions; and *v*_*m*_ is the average velocity of the incoming flow for the corresponding flow rate.
  $$\overline{{{\raise0.5ex\hbox{$\scriptstyle v$} \kern-0.1em/\kern-0.15em \lower0.25ex\hbox{$\scriptstyle {v_{m} }$}}}}$$ is the coefficient of the relative average flow velocity for each measurement point under the four operating conditions
  6 $$\overline{{\left( {\frac{v}{{v_{m} }}} \right)}} = \frac{{\sum\limits_{i = 1}^{i = 4} {\left( {\frac{{v_{i} }}{{v_{m} }}} \right)} }}{4}$$
  The relative average velocities in Fig. 6 are the arithmetic means of the relative velocities calculated for the four flow rate conditions.

Velocity field contour map of the test cross-section
  Given the boundary conditions, the internal velocity distribution contour maps of the circular bend pipe were obtained through numerical calculations for the four typical flow velocity conditions of the atypical circular bend pipe with a diameter of 0.4 m. Figure 5a–d show the velocity distribution contour maps for flow velocities of 14.904, 22.017, 26.224, and 29.076 m·s^−1^, respectively.

**Figure 6:**
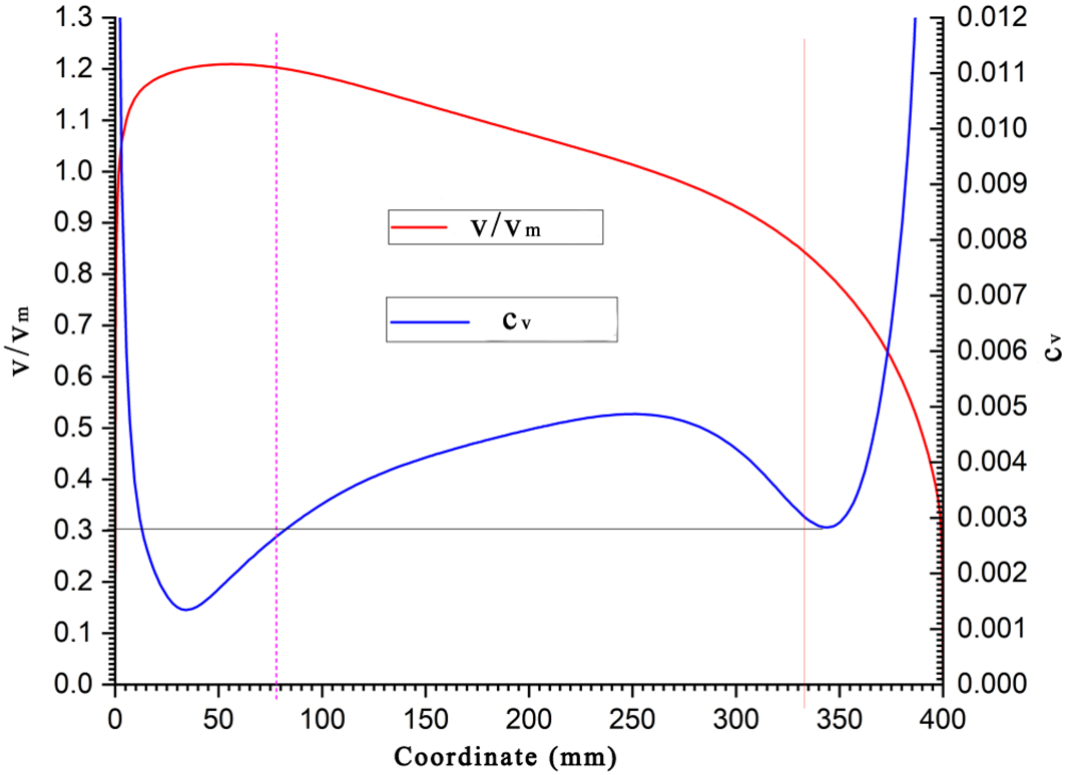
Locations for the average velocity and coefficient of variation measurements at the measurement points.
  For the coefficient of variation curve presented in Fig. 6, positions with higher coefficients of variation indicate a greater dispersion in relative velocity for the four flow conditions. This finding suggests a larger deviation from the mean in the velocity distribution along this column. Conversely, lower values imply that the relative velocities for the four flow conditions are closer to the average velocity. By evaluating the coefficient of variation, the two most stable measurement points can be determined in the stable region of the test cross section, as shown in Table 3 and Fig. 7.

**Table 3:** Coordinates of Measurement Points.

Coordinate z (m)	V/Vm	*C*_v_	Proposed measurement point number
0.078	1.2027	0.0026	1
0.330	0.8441	0.0030	2

**Figure 7:**
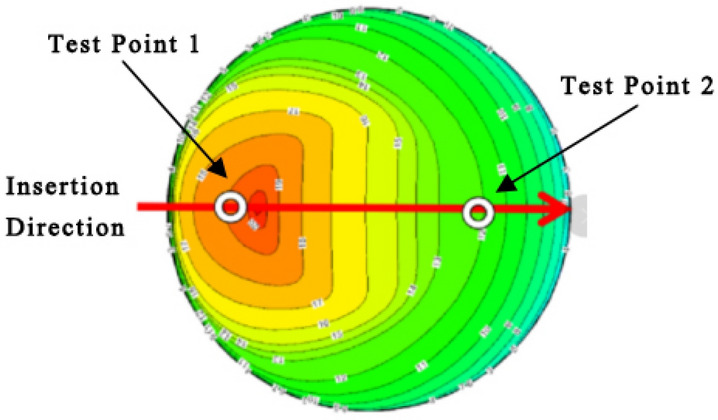
Schematic diagram of the measurement points distribution on the circular pipe test section.
Velocity difference flow rate calculation formula
  The weight coefficients with the pipeline flow rate are calculated as follows.
  7 $$\begin{gathered} Q = V_{m} \cdot A \hfill \\ V_{m} = \frac{{V_{2} }}{{(\frac{V}{{V_{m} }})_{2} }}\phi_{2} + \frac{{V_{1} }}{{(\frac{V}{{V_{m} }})_{1} }}\phi_{1} \hfill \\ \end{gathered}$$
  where $$({\raise0.5ex\hbox{$\scriptstyle V$} \kern-0.1em/\kern-0.15em \lower0.25ex\hbox{$\scriptstyle {V_{m} }$}})_{{\text{i}}}$$ represents the velocity coefficient at the sensor measurement point, *φ*_i_ represents the weight coefficient of each measurement point, and A is the test cross-sectional area. The calculations based on the above formula, using data from the test points recommended by the measurement rod as an example, are shown in Fig. 5. The coordinates of the two measurement points are determined, and the weight coefficients of these two points are calculated. The resulting calculations are as follows:
  $$\left( {C_{V} } \right)_{{2}} = 0.00{3}0$$
  $$\left( {C_{V} } \right)_{{1}} = 0.00{26}$$

The undetermined coefficients is calculated as follows:

8 $$1 = \frac{\phi }{{{0}{\text{.0026}}}} + \frac{\phi }{{{0}{\text{.0030}}}}$$

The solution is φ = 0.0014. The weight coefficients for each proposed measurement point were also calculated: 0.53 for measurement point 1 and 0.47 for measurement point 2.

The velocity is then calculated as follows:

9 $$V_{m} = \frac{{V_{1} }}{1.2027} \times 0.53 + \frac{{V_{2} }}{0.8441} \times 0.47$$

Thus, the flow rate calculation formula can be derived as follows:

10 $$Q = V_{m} \times A = V_{m} \times 3.14 \times 0.2^{2} = 0.1256V_{m}$$

The flow rate in atypical circular pipes can be measured using the differential pressure method. The motion of gas in these pipes follows the law of constant velocity momentum, and the gas movement within atypical circular pipes is an axisymmetric potential flow. As shown in Fig. 7, when the flow rate in the pipe to be measured is Q, the pressures at test points 1 and 2 are P_1_ and P_2,_ and the velocities are *v*_1_ and *v*_2_. Neglecting local losses and according to Bernoulli’s equation, the pressure difference between two points when the pipeline flow rate is Q is

11 $$\Delta P = \frac{{P_{1} }}{\rho g} - \frac{{P_{2} }}{\rho g} = \frac{{v_{2}^{2} }}{2g} - \frac{{v_{1}^{2} }}{2g}$$

Similarly, when the flow rate of the model pipeline is Q`

12 $$\Delta P{\prime} = \frac{{P_{1} ^{\prime}}}{\rho g} - \frac{{P_{2} ^{\prime}}}{\rho g} = \frac{{v_{2} ^{{\prime}{2}} }}{2g} - \frac{{v_{1} ^{{\prime}{2}} }}{2g}$$

According to the law of similarity in flow fields,

13 $$\frac{{v_{1} ^{\prime}}}{{v_{1} }} = \frac{{v_{2} ^{\prime}}}{{v_{2} }} = \frac{Q^{\prime}}{Q} = C$$

Therefore,

14 $$v_{1} ^{\prime} = Cv_{1} ,\;v_{2} ^{\prime} = Cv_{2} ,\;Q^{\prime} = CQ$$

Substituting v1` and *v*_2_` into the equation yields the following:

15 $$\Delta P{\prime} = C^{2} \frac{{v_{2}^{2} - v_{1}^{2} }}{{2{\text{g}}}} = C^{2} \Delta P$$

16 $$\frac{{\Delta P{\prime} }}{\Delta P} = C^{2} ,\;\frac{Q^{\prime}}{Q} = C^{2}$$

This substitution then yields

17 $$Q = \frac{{Q{\prime} }}{{\sqrt {\Delta P{\prime} } }} \times \sqrt {\Delta P} = \frac{{Q{\prime} }}{{\sqrt {\frac{{v_{2}{\prime}^{2} - v_{1}{\prime}^{2} }}{{2{\text{g}}}}} }} \times \sqrt {\frac{{v_{2}^{2} - v_{1}^{2} }}{2g}} = K\sqrt {\frac{{v_{2}^{2} - v_{1}^{2} }}{2g}}$$

*K* is the flow coefficient, which, based on numerical analysis and using Eqs. (11) and (17), can be fitted to determine that the flow coefficient *K* equals 2432.

## Experimental validation of the flow meter installation position in atypical circular bent pipes

### Chuan Yi flowmeter gas standard device

The main components of the negative pressure method critical flow nozzle gas flow standard device include a vacuum air source system, a critical flow Venturi nozzle metering section, a calibration pipeline system, a power air source system, standards and pipe sections, and an automatic control system. During operation, air flows through the calibration pipe section and the flowmeter being calibrated, passing through the critical flow Venturi nozzle assembly, then through the vacuum generation system, and finally back into the atmosphere.

**Figure 8:**
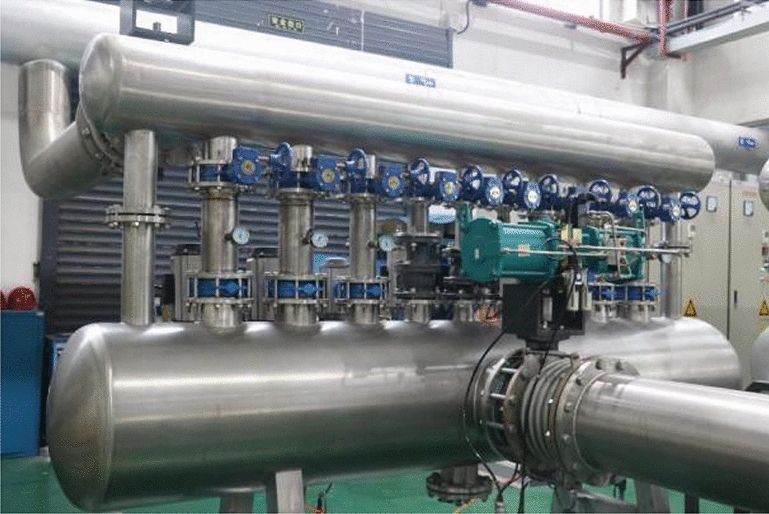
Vacuum air supply system.

**Figure 9:**
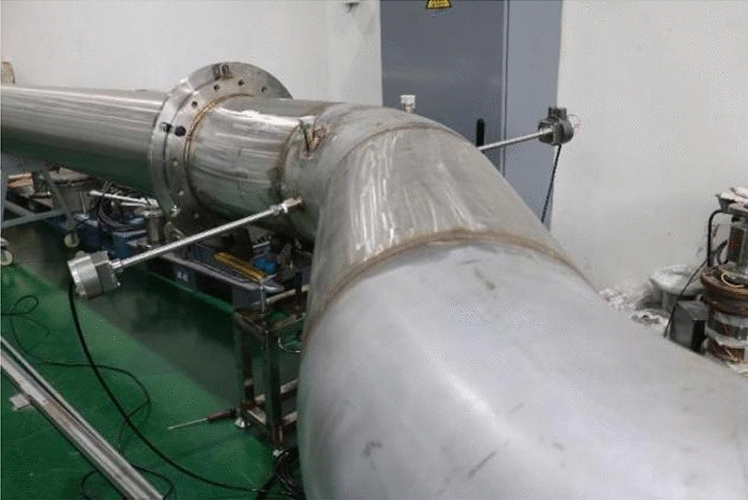
Pipeline system.

The mass flow rates of the gas passing through the critical flow nozzle and the tested flowmeter are the same. Under stable conditions, the microcomputer system automatically collects and processes the air stagnation pressure and stagnation temperature before the critical flow nozzle, calculates the standard gas mass flow and cumulative flow, and, according to relevant calibration procedures, compares and calculates it against the output of the tested flowmeter. This process determines the basic error and repeatability error of the tested flowmeter at different flow points, thus completing the calibration of the gas flowmeter. This experiment was conducted on a circular bent pipe with a diameter of 0.4 m, and the corresponding experimental apparatus was designed for validation, as shown in Figs. 8 and 9, to verify the differences between the flow field in the circular bend pipe and the simulation calculations.

### Design of the test section

**Figure 10:**
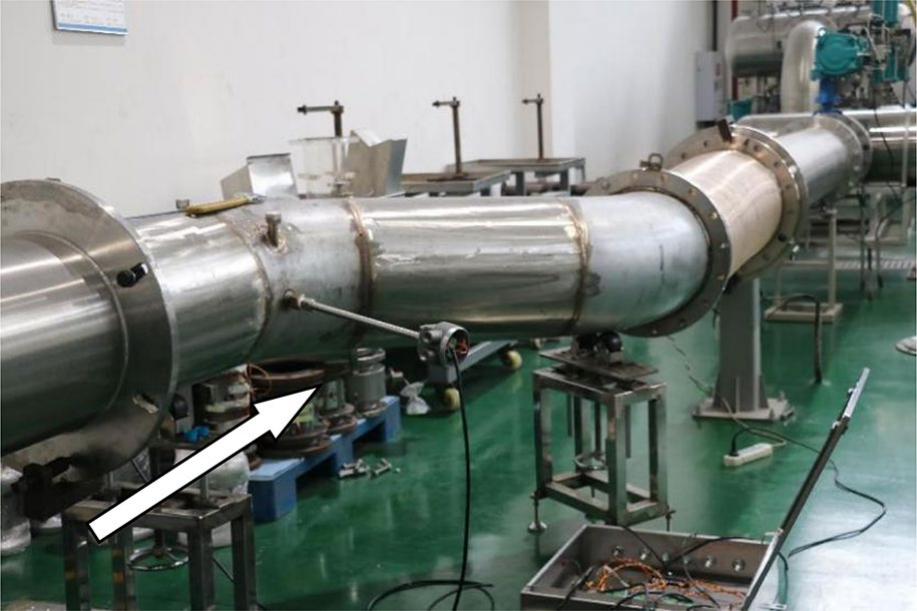
Physical image of the asymmetric circular pipe test section.

Following the design drawings, an atypical circular pipe test model was fabricated, as shown in Fig. 10. The model was constructed using A2-type steel to ensure the stability of the atypical circular pipe during flow. The sections were connected using flanges, with rubber gaskets laid at the joints to ensure airtightness during flow. The installation angle γ of the model was maintained at γ ≤ 1°, meeting the basic requirements of the experiment.

### Experimental method

The fabricated atypical circular pipe test model was tightly connected to the upstream section of the wind tunnel using flanges in the order of the test pieces, with rubber gaskets sealing between each piece to ensure the airtightness of the entire experimental model. Subsequently, the velocity testing equipment and displacement system were arranged on one side of the wind tunnel. A thermal mass flow meter was installed, and the stability of the testing system and the range of the displacement mechanism were checked in preparation for the experiment.

1. Two single-point insertion probes were calibrated on the DN100 device. Based on the simulation results, the relative velocity at the measurement points approached 1.3 times the pipeline’s average flow velocity (upper limit 30 m s^−1^), with the maximum flow velocity for calibration set to 40 m s^−1^.
2. A bent pipe fixture was installed on the DN400 gas standard device pipeline.
3. The two single-point insertion probes were installed onto the bent pipe fixture, with measurement point 1 inserted at 0.078 m and measurement point 2 at 0.330 m.
4. The entire machine was calibrated at 5% (1.5 m s^−1^), 25% (7.5 m s^−1^), 50% (15 m s^−1^), 75% (22.5 m s^−1^), and 100% (30 m s^−1^) of the upper limit range of 30 m/s, and the calculated errors were noted.

**Figure 11:**
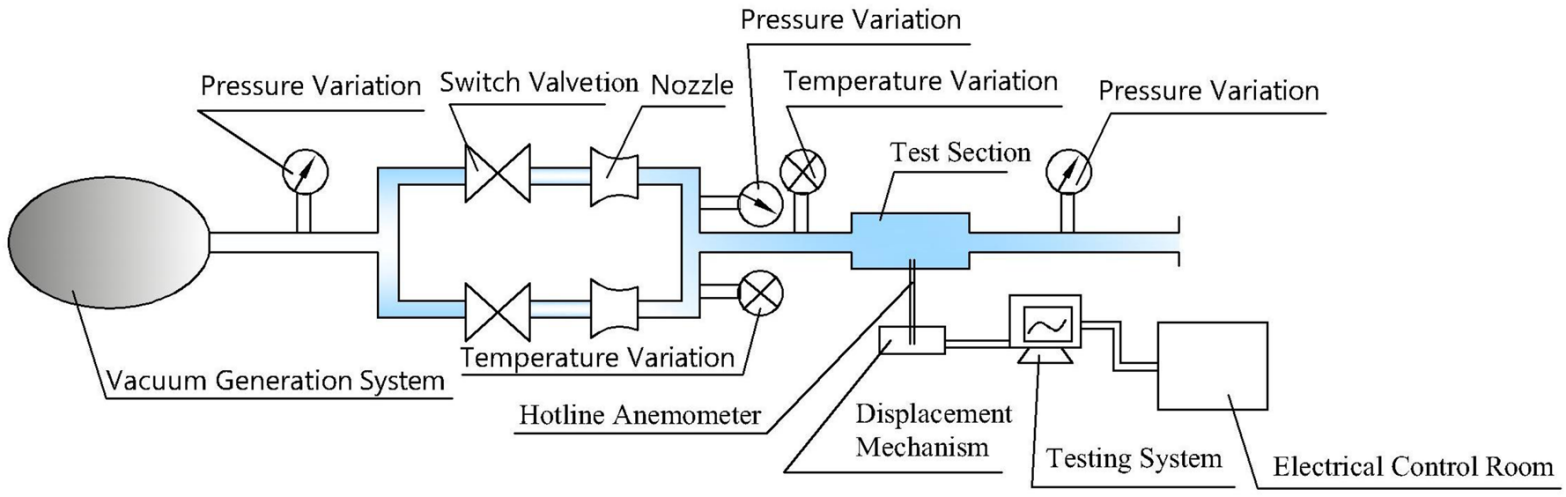
Experimental flowchart for the asymmetric circular bend.

Next, the tests for the four experimental conditions were conducted sequentially according to the frequency adjustment device and speed calibration device of the atypical circular pipe. The incoming wind speed generated by the circular pipe fan was controlled by adjusting the fan speed with a precision of 0.3%. The diagram of the experimental process is shown in Fig. 11.

## Error analysis

Using the calculation from Eq. (17), the statistical results shown in Table 4 were obtained. By comparing the calculated flow rate with the standard flow rate of the wind tunnel, the reference error was determined. The minimum error was 0.195%, and the maximum error was 0.625%. These results, within the range of these errors, meet the requirements for industrial applications.

**Table 4:** Verification of the error results for the computational formulas.

Operating condition	Flow point (%Q_max_) ($)	Velocity m s^−1^	Standard flow rate m^3^ h^−1^	Meter flow rate m^3^ h^−1^	Indicated value error (%RS)	Reference error (%FS)	Permissible error (%FS)
1	25	14.904	3365.3921	3374.5823	1.321	0.322	0.744
			3365.4858	3432.6770			
			3365.4405	3422.4030			
2	50	22.017	6714.3185	6780.3400	0.401	0.195	0.987
			6713.9599	6662.6235			
			6714.3939	6780.4245			
3	75	26.224	9669.8964	9727.3312	0.892	0.625	1.201
			9669.0948	9756.7293			
			9669.5682	9783.1275			
4	100	29.076	13,366.2855	13,462.8621	0.436	0.423	1.469
			13,364.6853	13,397.4665			
			13,366.1036	13,411.6855			

## Conclusion

This paper discusses a method to measure the flow in atypical circular bent pipes with a diameter of 0.4 m. The fundamental approach is to apply CFD for numerical analysis to obtain the distribution of flow velocities in the central area of the test cross section within the atypical circular bent pipe. This process established the optimal placement for installing a thermal mass flow velocity meter on the test cross section, leading to the formulation of a two-point method velocity calculation formula across the diameter line and, consequently, the determination of flow rates within ventilation ducts.

1. The flow field structure is similar under different flow rate conditions, with high-speed and low-speed areas on both sides of the flow field and a stable velocity distribution area in between.
2. A criterion based on the coefficient of variation was proposed to determine the optimal placement for installing the thermal mass flow velocity meter. The coordinates for the placement of the two sensors are 0.078 m and 0.330 m.
3. The measurement accuracy of the two-point method thermal mass flow velocity meter was verified. The calculated formula values were compared with the standard flow rate of the pipeline, and the reference error did not exceed 0.625%, meeting engineering requirements.

Through these experiments and analyses, this paper validates the feasibility of the method and demonstrates the practical application value of flow meters in measuring airflow in nuclear power plant ventilation ducts. This approach is not limited by the arrangement of ventilation ducts and can be broadly applied in ventilation duct systems.

## Data Availability

The datasets generated during and/or analysed during the current study are available from the corresponding author upon reasonable request.
